# Replication of Leaf Surface Structures on Flat Phosphor-Converted LEDs for Enhanced Angular Color Uniformity

**DOI:** 10.3390/mi15111399

**Published:** 2024-11-20

**Authors:** Bing-Mau Chen, Chiu-Hsiang Chen, Shang-Ping Ying, Yu-Kang Chang

**Affiliations:** 1 Department of Semiconductor and Electro-Optical Technology, Minghsin University of Science & Technology, 1, Xinxing Road, Xinfeng, Hsin-Chu 30401, Taiwan; bmchen@must.edu.tw (B.-M.C.); th6636@must.edu.tw (C.-H.C.); 2 Department of Medical Research, Tungs’ Taichung MetroHarbor Hospital, Taichung City 43503, Taiwan; t12193@ms3.sltung.com.tw; 3 Department of Post-Baccalaureate Medicine, College of Medicine, National Chung Hsing University, Taichung 40227, Taiwan; 4 Department of Nursing, Jenteh Junior College of Medicine, Nursing and Management, Miaoli 35664, Taiwan

**Keywords:** phosphor-converted LEDs, angular color uniformity, leaf surface microstructure, imprinting

## Abstract

We explored the use of biomimetic structures, including those that mimic leaf structures, to enhance the angular color uniformity of flat phosphor-converted light-emitting diodes (pcLEDs). The distinct microstructures found on natural leaf surfaces, such as micro-scale bumps, ridges, and hierarchical patterns, have inspired the design of artificial microstructures that can improve light extraction, scattering, and overall optical performance in LED applications. The effects of these leaf surface microstructures on the phosphor layer of flat pcLEDs were evaluated. An imprinting technique was employed to directly replicate the surface morphology structures from fresh plant leaves. The results indicated that this method provided excellent scattering capability and reduced the disparity in light output between blue and yellow light emissions from flat pcLEDs at various angles. Subsequently, uniform correlated color temperature in the flat pcLEDs was achieved, reducing the yellow ring effect. Furthermore, the availability of diverse wrinkle and surface patterns from a wide range of natural prototypes could reduce design costs compared with traditional mold fabrication, making the method suitable for application in mass production.

## 1. Introduction

**Figure 1:**
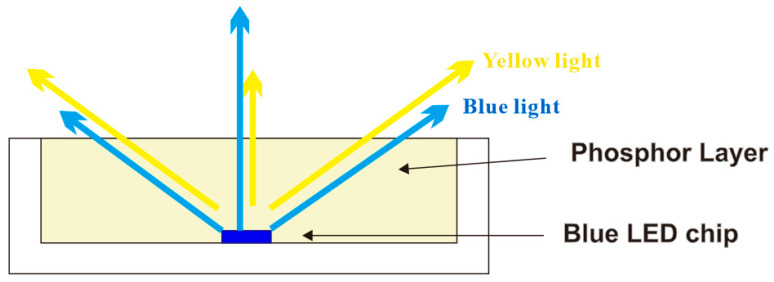
Schematic cross-sectional view of flat pcLED.

Light-emitting diodes (LEDs) are ubiquitous due to their high efficiency, long lifetime, low energy consumption, and environmental friendliness. There are two main approaches to generating white light with LEDs. One method involves mixing emissions from multiple colored LEDs, such as red, green, and blue, in suitable proportions. The other approach uses phosphors to downconvert blue LED emission into longer wavelengths, producing white light through color mixing. In general, phosphor-converted LEDs (pcLEDs) are predominantly used in the generation of white light because of their simple structure and low cost in mass production [1,2]. A portion of the blue light from a blue LED chip is converted by yellow phosphor to generate yellow light, and white light is produced by mixing the blue and yellow lights. However, specific problems still limit the use of pcLEDs in solid-state lighting applications, such as a low angular color uniformity (ACU) or the yellow ring effect [3,4]. The yellow ring effect can be attributed to the phosphor layer in the package. Longer excitation optical paths at larger angles lead to the conversion of more yellow light, and this additional yellow light at the perimeter of the LED reduces ACU, as shown in Figure 1. Furthermore, because of the large refractive index differences between the encapsulating materials and the air, considerable amounts of blue and/or converted yellow light are trapped within the package because of the total internal reflection effect. The trapped light either escapes from the side of the platelet or is reabsorbed and converted to heat, both of which reduce forward light emission. This phenomenon reduces lumen output and further exacerbates the yellow ring effect in pcLEDs.

Various studies have attempted to address the yellow ring effect or ACU, both of which are crucial indicators of the optical properties of high-quality LEDs [5,6,7,8,9,10]. One proposed method involved the use of a dome lens package, where hemispherical encapsulation with various phosphor layer geometries and locations were used to improve the ACU in pcLEDs. However, the special geometry and large volume of the dome lens made these devices difficult to miniaturize. Additionally, the need for planar encapsulation instead of dome lens packages has increased due to the current trend toward more compact package designs. Another effective method for achieving a low ACU involved the application of materials with a strong scattering capability to improve color uniformity and optimize the properties of pcLEDs [11,12]. Through the introduction of patterned structures on the encapsulation layer, color mixing can be optimized without substantially increasing manufacturing complexity or cost. Patterned structures, such as surface-textured structures on planar encapsulation surfaces, may provide an alternative method for improving the ACU of pcLEDs. A textured polydimethylsiloxane (PDMS) pattern can then be transferred using a simple imprinting technique to form a textured planar encapsulation. PDMS, a flexible, transparent silicone material, can be easily molded into various micro- and nano-scale structures, making it highly suitable for creating textured surfaces on planar encapsulation. Additionally, PDMS is highly compatible with roll-to-roll processing, allowing for the cost-effective mass production of large-area patterned films. PDMS films with microstructured surfaces are particularly beneficial for LEDs because they can be applied as encapsulation layers to substantially improve light scattering, thereby enhancing color uniformity. The light scattering effect from a surface-textured PDMS film has been verified to effectively eliminate the yellow ring effect of pcLEDs [13,14]. Additionally, the textured pattern created through the imprinting technique can be transferred directly onto the phosphor layer of pcLEDs [15,16,17]. Although various surface texturing methods have been investigated, some have been demonstrated to be insufficient in eliminating the yellow ring effect. A major challenge with the imprinting technique for pcLEDs is the high cost associated with designing and manufacturing molds with patterned structures. Additionally, innovative pcLED structures with textured surfaces need to be developed to ensure uniform color distribution.

In natural light–harvesting systems, green plants are crucial for transforming sunlight into chemical energy through photosynthesis. This essential process, primarily occurring in the leaves, is the main source of both oxygen production and organic matter synthesis on Earth. Over millions of years of evolution, plant leaves have developed highly specialized surface structures to optimize light absorption and adapt to varying environmental conditions. These surfaces are equipped with a diverse range of micro- and nano-structures, each designed to maximize photosynthetic efficiency by enhancing light capture and utilization. These intricate surface structures not only enhance photosynthetic efficiency by maximizing light absorption, they also influence the capture and scattering of light. Additionally, leaves from different plant species differ in their photosynthetic abilities, efficiencies, and surface structures, even when grown in similar natural environments. This diversity reflects adaptations to specific ecological niches and light conditions. The wide array of natural prototypes, such as micro-scale bumps, ridges, and hierarchical patterns, provides a valuable library of wrinkle and surface styles that can inform the design of artificial light–harvesting surfaces and covers. Numerous studies have investigated biomimetic structures in optics, focusing on how plant surfaces can inspire the development of innovative designs that enhance light management and improve performance in various optical applications [18,19,20]. By mimicking the complex micro- and nano-scale structures found in leaves, which are optimized by nature to manage light efficiently, researchers have developed surfaces that substantially improve light scattering, absorption, and extraction [21,22,23]. Thus, the use of biomimetic structures, including the mimicking of leaf structures, can enhance the color uniformity of pcLEDs. The distinct microstructures found on natural leaf surfaces, such as micro-scale bumps, ridges, and hierarchical patterns, can inform the design of artificial microstructures that can improve light extraction, scattering, and overall optical performance in LED applications.

In the present study, the effects of leaf surface microstructures on the phosphor layer of pcLEDs were evaluated. An imprinting technique was employed to replicate surface-textured structures directly from fresh plant leaves. This approach provided excellent scattering capability and reduced the variation in light output between blue and yellow light emissions from pcLEDs at various angles. Consequently, a uniform correlated color temperature (CCT) was achieved for pcLEDs, which reduced the yellow ring effect. Furthermore, the availability of diverse wrinkle and surface patterns from a wide range of natural prototypes would mean a reduction in design and fabrication costs, compared with traditional mold fabrication, making the method suitable for application in mass production.

## 2. Materials and Methods

**Figure 2:**
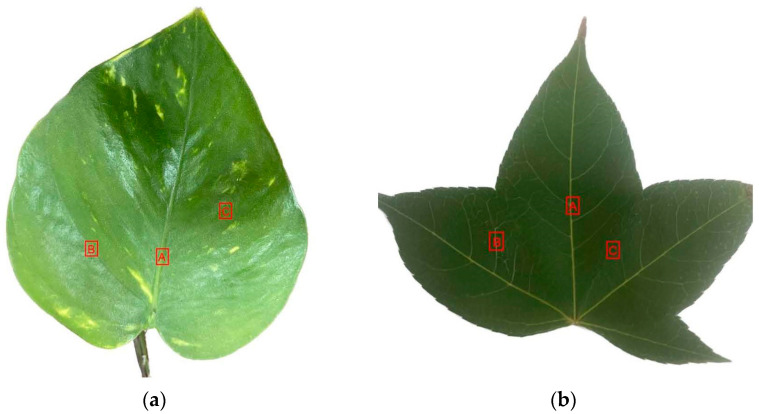
Photographs of the leaves used in the study. (**a**) *Epipremnum aureum* and (**b**) *Acer serrulatum*, with marked regions indicating areas selected for surface microstructure analysis.

In our experiments, leaf surface microstructures were applied onto the phosphor layer of pcLEDs. Specifically, these leaves were used as a master template to fabricate a patterned microstructure onto the phosphor layer of pcLEDs. For the use of leaf surface microstructures on pcLEDs to enhance angular color uniformity, selecting leaf structures of appropriate size is essential. Therefore, common plants with medium-sized leaves from Northern Taiwan were chosen for this study. The size and morphology of the surface microstructures were also key factors for effective replication. Two different plants from Northern Taiwan, *Epipremnum aureum* and *Acer serrulatum*, were selected on the basis of their surface microstructure sizes (20–50 μm) and distinct morphological features. To investigate the influence of varying leaf surface microstructures on optical properties, we selected three distinct regions from the same leaf of two different plants for imprinting fabrication, as shown in Figure 2a,b. Region A refers to the part of the leaf where the petiole connects the leaf to the stem. Region B represents the area around the leaf veins, where the vascular tissue is most prominent. Region C is the section of the leaf that is free from both the petiole and veins, consisting of the broader, uninterrupted surface of the leaf blade. These regions were chosen to explore the potential differences in microstructure between areas with varied surface morphologies. By selecting regions that include structural differences, such as the presence of vascular tissue (Region B) and areas with smooth, uninterrupted leaf surfaces (Region C), we can better understand how different microstructural features affect light absorption, reflection, and other optical properties.

**Figure 3:**
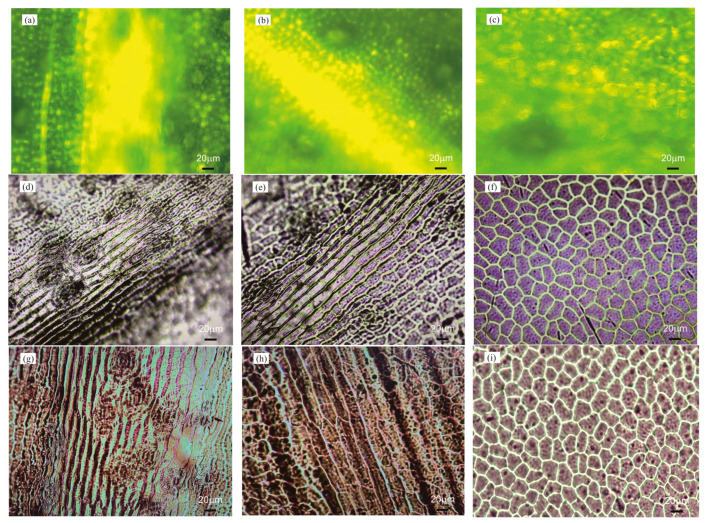
Optical microscope images of the surface morphologies of (**a**–**c**) Regions A, B, and C from *Epipremnum aureum*, (**d**–**f**) their respective inverse UV-curable resin replicas, and (**g**–**i**) their respective positive replicated PDMS films.

**Figure 4:**
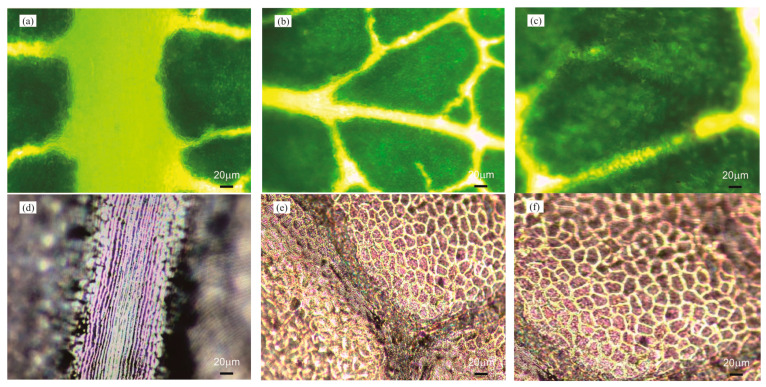

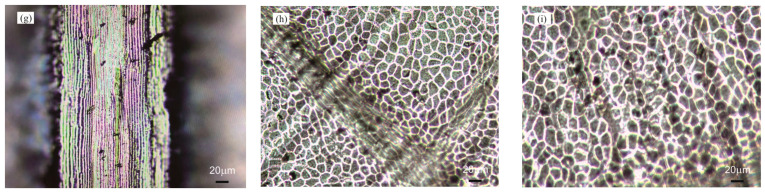
Optical microscope images of the surface morphologies of (**a**–**c**) Regions A, B, and C from *Acer serrulatum*, (**d**–**f**) their respective inverse UV-curable resin replicas, and (**g**–**i**) their respective positive replicated PDMS films.

**Figure 5:**
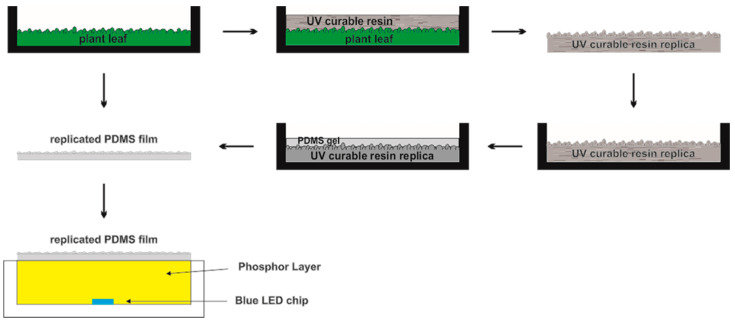
Schematic demonstrating the procedure used to produce lead surface microstructure on pcLEDs.

Figure 3a–c and Figure 4a–c present photographs of the original *Epipremnum aureum* and *Acer serrulatum* leaf surfaces from regions (A), (B), and (C), which were captured using a Nikon Eclipse Ci-S upright clinical microscope. To maintain their natural surface features and avoid dehydration artifacts, the leaves were freshly picked immediately before each replication process. The fresh leaves were cleaned with ethanol and dried with pressurized air to remove any moisture. The selected plant leaf was cut into small pieces of approximately 2 cm × 2 cm, and a piece was fixed to a clean Petri dish using double-sided adhesive. To replicate surface microstructures, a polyurethane acrylate (GC251, Everwide Chemical Co., Yunlin County, Taiwan) ultraviolet (UV)-curable resin was used. The UV-curable resin was slowly poured onto the selected leaf piece and cured under UV light. After the UV-curable resin was fully cured, it was peeled off, revealing a high-fidelity inverse replica of the *Epipremnum aureum* and *Acer serrulatum* leaves’ structural membrane, as shown in Figure 3d–f and Figure 4d–f. Subsequently, the leaf surface microstructures were fabricated on a PDMS film by using the first inverse UV-curable resin replica as an imprint stamp. Two-component PDMS gel (Sylgard 184, Dow Corning, Midland, MI, USA), with a weight ratio of 10:1 (elastomer to crosslinker), was uniformly mixed and slowly poured onto the inverse UV-curable resin replica, coating the Petri dish. The sample was heated in an oven at 120 °C for 1 h to cure the thin PDMS film, and the replicated PDMS film was peeled off from the first inverse UV-curable resin replica. The leaf microstructures were then reversely transferred from the first inverse UV-curable resin replica onto the surface of the replicated PDMS film. Figure 3g–i and Figure 4g–i depict the microstructures formed on the high-fidelity replicated PDMS films of *Epipremnum aureum* and *Acer serrulatum*. The photographs reveal that the positive microstructures formed on the replicated PDMS films accurately reproduced the leaf surface topology. To ensure that the optical characteristics for different plants were comparable, all replicated PDMS films were fabricated with a thickness of approximately 0.4 mm. To apply the leaf surface microstructures on the phosphor layer of pcLEDs, the same blue LED chips and the same amount of phosphor mixed with silicone encapsulant were used in this study. Additionally, PDMS was used as the encapsulant for pcLEDs. The blue LED chips, with a chip size of 1 mm × 1 mm and a wavelength of 450 nm, were mounted on the commercial plastic lead-frame package. A commercial YAG:Ce phosphor (YAG4EL, Intematix, Fremont, CA, USA) was dispersed in PDMS gel, uniformly stirred, and dispensed to fill up the lead-frame package cup. After the phosphor layer was cured in an oven at 120 °C for 1 h, the prepared replicated PDMS film was covered on the top surface of the pcLEDs. The schematic process flow for fabricating leaf surface microstructures on the phosphor layer of pcLEDs is shown in Figure 5.

## 3. Results and Discussion

**Figure 6:**
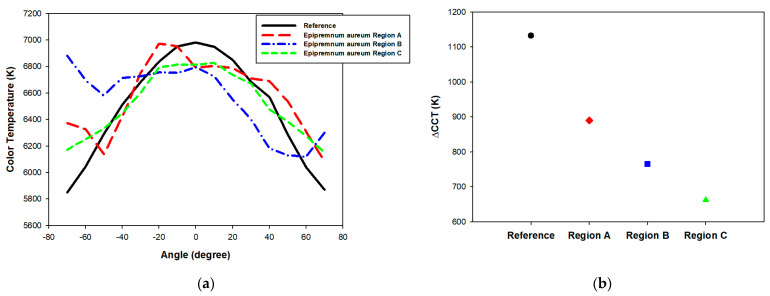
(**a**) Angular CCT and (**b**) ΔCCT of pcLED with and without *Epipremnum aureum* surface microstructures applied to phosphor layer.

**Figure 7:**
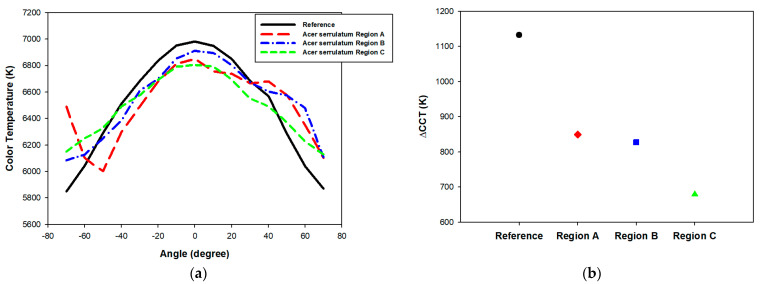
(**a**) Angular CCT and (**b**) ΔCCT of pcLED with and without *Acer serrulatum* surface microstructures applied to phosphor layer.

The angular CCT uniformity of LEDs is a crucial parameter for evaluating the quality of light output, particularly in applications requiring high ACU. ACU can be quantitatively assessed using the ΔCCT metric, which is defined as the difference between the maximum and minimum CCT values observed within an angular range from −70° to 70°. In this study, a stepping motor controller (08TMC-2U, Unice E-O Services Inc., Taoyuan, Taiwan) was used to control the rotary stepper motor, in conjunction with a compact spectrometer (Ocean SR2, Ocean Optics Inc., Orlando, FL, USA). An optical fiber with a 1000-micrometer core diameter was connected to the spectrometer at one end, while the other end collected light emitted from the pcLED at various angles, to calculate the CCT. The fiber entrance was positioned 1 m away from the pcLED, aligned with the center of the rotary stepper motor. LabVIEW was employed to automate the measurement of angular CCT at 5-degree intervals. We investigated the angular-dependent CCT characteristics of pcLEDs with distinct regions (Region A to Region C) of *Epipremnum aureum*. The selection of regions for imprinting fabrication from the area covering the leaf petiole (Region A), leaf veins (Region B), and the broader, uninterrupted surface of the leaf blade (Region C) was methodically designed to investigate the impact of distinct microstructural features on the optical properties of pcLEDs. Figure 6a presents the angular CCT of the pcLEDs with various *Epipremnum aureum* surface microstructures applied to the phosphor layer. As depicted in Figure 6b, these microstructures considerably enhanced color uniformity, reducing ΔCCT ranges approximately from 1130 K to 660 K compared with the reference sample without surface microstructures. This improvement indicates that the use of an *Epipremnum aureum* surface microstructure on pcLEDs effectively mitigates angular color variations by enhancing the scattering of emitted light, resulting in a more uniform distribution of blue and yellow light emissions across multiple viewing angles. Furthermore, Region A, where the petiole connects the leaf to the stem, exhibits a dense and irregular microstructure influenced by the thickness of the petiole. This structure likely contributes to highly non-uniform light scattering, resulting in asymmetric angular color uniformity. As shown in the angular CCT plot, the CCT curve of Region A displays significant fluctuations, indicating asymmetric, but relatively low, angular color uniformity. In contrast, Region B, which encompasses the area around the leaf veins with prominent vascular tissue influenced by the thickness of the veins, presents a more organized yet varied microstructure. The corresponding CCT curve for Region B is more symmetric compared to Region A, though it still shows some degree of angular variation, indicating moderate improvement in angular color uniformity. Moreover, Region C, characterized by its smooth and uninterrupted surface, has the most homogeneous microstructure. This results in the highest angular color uniformity, as reflected by the symmetric CCT curve of Region C. The smooth microstructure effectively mitigates light scattering variations, providing consistent CCT values across angles. This suggests that regions with smoother and more uniform microstructures are more effective in achieving consistent CCT and mitigating the yellow ring effect in pcLEDs. Figure 7a illustrates the pcLEDs with various *Acer serrulatum* surface microstructures (Region A to Region C) on the phosphor layer. A reduction in ΔCCT ranges, approximately from 1130 K to 680 K relative to the reference sample without surface microstructures, was identified, as shown in (Figure 7b). This improvement in ΔCCT with the application of leaf surface microstructures on pcLEDs can be attributed to the ability of these microstructures to scatter blue light more effectively at larger angles, leading to a more uniform mix of blue and yellow light emissions. The larger ΔCCT identified in the pcLEDs without leaf surface microstructures can primarily be attributed to the differing emission patterns of the LED chips and the yellow phosphor, resulting in lower blue light intensity at oblique angles. Consequently, the color temperature is higher in the normal direction than at wider angles. By contrast, the addition of leaf surface microstructures enhances the scattering of blue light, thereby reducing CCT variations and improving color uniformity. Additionally, the surface structure near the petiole (Region A), defined by a dense and fibrous micro-pattern, plays a role in light scattering, contributing to a moderate yet asymmetric reduction in ΔCCT. However, the color control in this region remains less effective compared to the other areas due to the irregularity of the structure. In contrast, Region B, located around the leaf veins, features a more structured yet varied micro-pattern that enhances light scattering. This leads to an asymmetric, but noticeable, improvement in color uniformity. Region C, which covers the broader, smoother area of the leaf, stands out with its highly homogeneous microstructure, providing the most efficient light scattering. As a result, this region achieves the greatest reduction in ΔCCT and exhibits the highest angular color stability. The smoother surface of Region C minimizes variations in light scattering, ensuring more consistent color performance across different angles. These findings suggest that regions with smoother and more regular microstructures are particularly effective in stabilizing the CCT and reducing color inconsistencies in pcLEDs.

**Figure 8:**
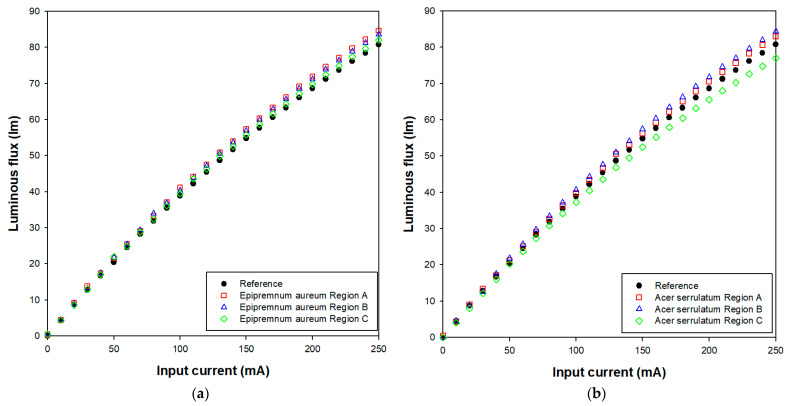
Optical power according to the injection current of pcLED with and without (**a**) *Epipremnum aureum* and (**b**) *Acer serrulatum* surface microstructures applied to phosphor layer.

To further understand the optical performance of the pcLEDs with different leaf surface microstructures, the current-dependent luminous flux was measured. The optical characteristics of the pcLEDs were evaluated using a source meter (PSM-3004, Good Will Instrument Co., New Taipei City, Taiwan), a photodetector connected to an optometer (S380, OSI Optoelectronics, Hawthorne, CA, USA), and a compact spectrometer (Ocean SR2, Ocean Optics Inc., Orlando, FL, USA) integrated with a 30 cm integrating sphere. As shown in Figure 8a, the luminous flux of the pcLEDs with various *Epipremnum aureum* surface regions increased by 1.8% to 5% at a driving current of 250 mA; the luminous flux of the pcLEDs with various *Acer serrulatum* surface regions increased by 2.7% to 4.4% at a driving current of 250 mA, except Region C (as shown in Figure 8b). Specifically, Region A shows the highest increase, followed by Region B and Region C. This enhancement in luminous flux can be attributed to the ability of the leaf surface microstructures to improve light extraction efficiency by scattering light more effectively at different angles, reducing internal reflection losses. The surface structure near the petiole (Region A), characterized by a dense and fibrous micro-pattern, plays a significant role in increasing light scattering, leading to the highest luminous flux increase. This structure directs more light outward, preventing it from being trapped inside the phosphor layer, and demonstrates the most notable improvement in luminous output. In contrast, Region B, located around the leaf veins, features a more structured yet varied micro-pattern. This arrangement promotes enhanced light scattering, leading to a noticeable, but slightly less pronounced, increase in luminous flux compared to Region A. Meanwhile, Region C, characterized by its smoother and more homogeneous surface, provides the lowest but still significant improvement in luminous flux. The smoother microstructures in this region may be less effective at scattering light compared to the more complex micro-patterns in Regions A and B. The overall increase in luminous flux across all regions indicates that leaf surface microstructures, especially those with more complex and fibrous patterns, play a critical role in enhancing the optical performance of pcLEDs by improving light extraction and scattering, thereby boosting luminous output. Although the current-dependent luminous flux of the pcLEDs with *Acer serrulatum* surface microstructures (Regions A to C) follows a similar trend to those with *Epipremnum aureum*, distinct differences can be observed. As shown in Figure 7b, only Regions A and B of *Acer serrulatum* show an enhancement in luminous flux compared to the reference pcLED. Region B exhibits the highest improvement, followed by Region A, with both regions enhancing the luminous output. In contrast, Region C presents a lower luminous flux than the reference sample, deviating from the trend observed in the other regions. This reduction in luminous flux for Region C may be due to imperfections in the inverse UV-curable resin replicas and the replicated PDMS films of *Acer serrulatum*, produced via the imprinting technique. These surface irregularities likely diminish the light extraction efficiency by disrupting the light scattering process, resulting in a lower luminous output than the reference pcLED, despite the smoother and more homogeneous microstructure of Region C.

## 4. Conclusions

In the present study, the effects of leaf surface microstructures on the phosphor layer of phosphor-converted LEDs (pcLEDs) were evaluated. An imprinting technique was employed to directly replicate surface morphology structures from fresh plant leaves. This innovative method provided excellent light scattering capabilities and effectively minimized the differences in light output between blue and yellow light emissions at various angles. Through the application of this method, pcLEDs with uniform CCT were achieved, substantially reducing the yellow ring effect. In addition, different regions of the leaf surface exhibited varying effects on color distribution. The surface near the petiole provided moderate light scattering but less effective color control, while the area around the veins enhanced scattering and improved color distribution. The smoother region offered the best light scattering and reduction in ΔCCT, resulting in consistent color performance across the viewing angles. These varying microstructures contributed to more stable color output and better performance in pcLEDs. Overall, the present study presents a promising, cost-effective method for enhancing the optical performance and manufacturing efficiency of pcLEDs, paving the way for future advancements in LED technology. By utilizing diverse wrinkles and surface patterns derived from a wide range of natural prototypes, this method reduces design and fabrication costs compared with traditional mold fabrication techniques, making it highly suitable for application in mass production.

## Data Availability

The data presented in this study are available on request from the corresponding author.
